# The Value of Tranexamic Acid in Reducing Blood Loss following Hip Reconstruction in Children with Cerebral Palsy

**DOI:** 10.1155/2015/827027

**Published:** 2015-11-17

**Authors:** I. Majid, S. Alshryda, B. Somanchi, E. Morakis, A. Foster

**Affiliations:** Royal Manchester Children Hospital, Central Manchester University Hospitals, Oxford Road, Manchester M13 9WL, UK

## Abstract

This is a retrospective study of 51 consecutive hip reconstructions in children with cerebral palsy performed between 2011 and 2013. Tranexamic acid (TXA) was used in 14 hip reconstructions only. Transfusion rate was higher, postoperative Hb was lower, and patients stayed longer in the TXA group. This did not reach a statistical significance (*P* = 0.75, 0.5, and 0.71, resp.). More than half of the patients who had TXA underwent bilateral hip reconstructions in comparison with 27% only in the non-TXA group. Bilateral hip reconstructions mean more surgery, more blood loss, and more blood transfusion. The patients who had TXA were significantly more disabled as evident by the higher proportions of patient with worse GMFCS levels. Although we have not been able to demonstrate the value of TXA in reducing blood loss and transfusion rate in children with CP who underwent hip reconstruction, it is hoped that an interest in exploring the value of TXA in paediatric orthopaedic surgery is generated. Ideally this should be explored further in an adequately powered, randomised controlled trial where risk of bias is minimized.

## 1. Introduction

Cerebral palsy (CP) is caused by an injury to the immature brain usually occurring during or shortly after birth. Although all functions of the brain may be affected, the motor function is usually the most vulnerable [1]. The gross motor function classification system (GMFCS) categorises the functional capabilities of children with CP into 5 levels (Table 1) [2].

**Figure 1:**
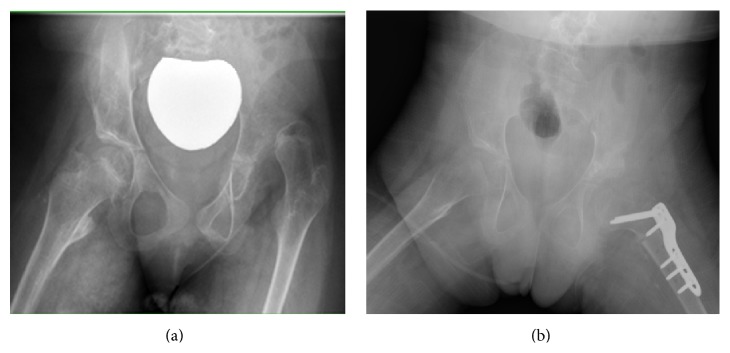
Preoperative and postoperative X-rays of a dislocated hip in a child with cerebral palsy. The left X-ray showed a dislocated left hip in a child with CP. The acetabulum has become shallow and is not covering the femoral head any more. There is a valgus deformity of the proximal femur. The postoperative pictures showed that the hip is reduced, the proximal femoral deformity is corrected (varus derotation corrective osteotomy), and the acetabulum is corrected by pelvic Dega osteotomy.

The initial treatment usually involves a combination of interventions such as medicines, braces, and adaptive and assistive equipment; however, surgery is sometimes warranted to control symptoms and maintain an optimum level of function and appearance. Hip joint dislocation is a common problem in children with CP that can cause significant pain and interference with personal care and hygiene. Surgical hip reconstruction reduces the hip joint through soft tissue releases and bony cuts of the femur and/or pelvis (Figure 1). Blood loss and subsequent blood transfusion are a normal consequence of hip reconstruction [3, 4].

Postoperative anaemia has been shown to impede functional ability and therefore delay discharge in this patient group. However allogeneic blood transfusion is associated with risks for the recipient (haemolysis, infection, immunosuppression, transfusion-related acute lung injury, and even death). The risk of postoperative wound infection correlates with the amount of transfused allogeneic blood and has significant cost implications. Thus surgeons aim to minimise blood loss at surgery.

There has been a recent surge in the use of tranexamic acid (TXA) in trauma and orthopaedic practice. TXA belong to a group of agents called “the antifibrinolytics” which has been used successfully to reduce blood loss. A Cochrane review scrutinised 252 trials that had used antifibrinolytics in elective surgery: 60 trials evaluated TXA. The reviewers concluded that antifibrinolytics are effective in reducing blood loss, the need for allogeneic red cell transfusion, and the need for reoperation due to continued postoperative bleeding after elective surgery [5]. Numerous other studies have confirmed the effectiveness of TXA in reducing blood loss and transfusion requirements in total joints replacements and spine surgery [6–12]. However, there is very little published work on the use of TXA in paediatric orthopaedic practice.

In a multicenter, retrospective review of 84 patients with CP who underwent spinal deformity correction [13], the use of TXA was associated with less blood loss and length of stay. Very few surgeons use TXA in hip reconstruction surgery for children with CP and, to the best of our knowledge, there is no available report on its effectiveness and safety profile in this group of patients.

## 2. Methods

A retrospective observational study design, including 51 consecutive hip reconstructions performed between 2011 and 2013, was used to evaluate the use of tranexamic acid (TXA). The study was approved by the Institution's Audit Department (reference number 4942). Medical records were reviewed for age, date of birth, gender, medications, weight at surgery, blood loss in theatre, and the need for transfusion. The latter was cross-checked with the blood bank data base for confirmation. The preoperative laboratory results of complete blood count including hemoglobin (Hb), platelets, and haematocrit (Hct) were recorded. The estimated blood volume (EBV) was estimated from the following formula: (EBV = 80 mL *∗* body weight in kg). The estimation of red blood cell volume (RBCV) at preoperative Hct is as follows: RBCV_preop_ = EBV ×  Hct_preop_. The estimation of red blood cell volume (RBCV) at postoperative Hct is as follows: RBCV_Postop_ = EBV ×  Hct_Postop_. The total blood loss is calculated from the fall in red blood cell volume: Blood loss = RBCV_diff_/Hct_Postop_ [14, 15].

Data cleaning and analysis were performed using SPSS 20. The data were checked for normality using the Kolmogorov-Smirnov test (KST) and the Shapiro-Wilk test (SWT). When substantial nonnormality was indicated (*P* < 0.05), then nonparametric estimate was provided alongside the parametric findings. Moreover, a bootstrap estimate was provided to explore robustness of estimates [16]. Continuous outcomes were analysed (blood loss, volume transfused, length of stay, haemoglobin and Haematocrit, and overall cost), using independent samples *t*-test. Categorical outcomes were analysed by Fisher's exact test (blood transfusion required, GMFCS levels, and complications). The study main outcome was transfusion rate with other measures considered supportive.

## 3. Results

Fifty-one hip reconstructions were performed in children with CP in our institute: 14 received TXA, 37 did not. The two groups were similar in age, weight, gender distribution, and the ASA Physical Status (the American Society of Anesthesiologists) (see Table 2). Baseline mean operative Hb levels and Hct levels were similar, as were proportions of patients taking nonsteroidal anti-inflammatory drugs (NSAID) and Epilim: these are implicated with increased surgical blood loss [17, 18]. Patients in the tranexamic acid group had a higher GMFCS level (78% versus 75%: *P* = 0.79), more combined pelvic and femoral osteotomies (78% versus 67%: *P* = 0.62), and more bilateral surgery (57% versus 27%: *P* = 0.06). Although none of these differences reached a statistical significance, the latter had come quite close. Bilateral hip reconstruction was performed in 57% of children in the TXA group in comparison to 27% in the other group (*P* = 0.06); thus the TXA group may have more severe underlying disease. There is a borderline significant different in the pattern of gross motor function classification system (GMFCS) between groups, despite no systematic trend by level (*P* = 0.79).

### 3.1. Blood Transfusion

The proportion of children receiving blood transfusion was 42.9% in the TXA groups versus 36.1% in the non-TXA group, a statistically nonsignificant difference (RD: 6.7%, 95% CI: −21.3% to 36.1%, *P* = 0.75) (Table 3).

#### 3.1.1. Blood Loss

The total blood loss was estimated using Gross's formula [14, 15]. The mean total blood loss was similar: TXA 969 mL versus no TXA 971 mL (RD: −2 mL, 95% CI: −536 mL to 531 mL, *P* = 0.99).

#### 3.1.2. Postoperative Hemoglobin, Haematocrit, and Hb Drop

Hemoglobin (Hb) and hematocrit (Hct) were tested on postoperative day 2 unless there was a clinical need to do these earlier. Levels were similar and differences between the two groups were not statistically significant.

#### 3.1.3. Length of Stay

The duration of stay in hospital was similar between groups: TXA 8.17d versus no TXA 7.63 (difference: 0.54 d, 95% CI: −2.37 to 3.45), *P* = 0.71.

### 3.2. Adverse Events

There were 3 complications in the tranexamic acid group (2 fragility fractures and 1 paralytic ileus). The two fractures were treated nonoperatively in a resting cast. Two complications were recorded in the non-TXA group: one deep infection and a pressure sore. The former was treated with washout and antibiotic and the latter was treated with regular dressing. There were no reported thromboembolic events (see Table 4).

## 4. Discussion

This retrospective study was conducted to explore the transfusion rate in children with cerebral palsy who underwent hip reconstruction as a background work for a future trial. Blood transfusion rate is considered to be a reliable outcome measure and it is of great importance for patients, healthcare providers, and managers and hence it is chosen as the primary outcome for our study. A recent local audit showed that the our unit was 96% compliant with the national guidelines for blood transfusion [19, 20] supporting the findings and subsequent conclusions of this study. Out of 51 hip reconstructions, 13 patients (25.4%) received allogenic blood transfusion. Contrary to most studies [5, 11, 12] that used TXA, in children with CP and hip reconstruction, transfusion rate was higher, postoperative Hb was lower, and patients stayed longer in the TXA group. This did not reach a statistical significance (*P* = 0.75, 0.5, and 0.71, resp.). This may be a true finding that TXA does not reduce blood loss in this group of patients or it may be a type II error where the study did not demonstrate the real positive effect of TXA on blood transfusion for various reasons.

The table of baseline characteristics of the study populations showed significant selection bias which disfavours TXA. It is apparent that TXA was used when larger blood loss was expected. More than half of the patients who had TXA underwent bilateral hip reconstructions in comparison with 27% only in the non-TXA group. Bilateral hip reconstructions mean more surgery, more blood loss, and more blood transfusion. The patients who had TXA were significantly more disabled as evident by the higher proportions of patient with worse GMFCS levels. It is expected that these factors would mean more transfusion rates in the TXA group and nevertheless this was not the case. Similar can be said on length of stay where patients who received TXA stayed longer in hospital.

Eipe and Ponniah [14] showed that surgical blood loss was underestimated by 64% using clinical methods assessing blood soaked mops and gauze pieces and measuring blood lost to suction bottles and the vacuum drain. They recommended using a biochemical method based on Hct. In our study, total blood loss was estimated using Gross's formula [14, 15]. The difference was not clinically important and statistically was not significant.

There are several weaknesses to our study. It is a retrospective study where data were collected from medical records and hospital laboratory. Although this may undermine the accuracy of data, most primary outcomes (blood transfusion, HB level, and length of stay) are accurately and independently recorded in our center. The number of patients involved in our study is relatively small. It is estimated that 184 patients are required to have an 80% chance of detecting a 50% decrease in the transfusion rates of 36% in the non-TXA group at the 5% level. The rate of the type II error is closely related to the number of participants involved in a study (the study power).

In conclusion, although we have not been able to demonstrate the value of TXA in reducing blood loss and transfusion rate in children with CP who underwent hip reconstruction, it is hoped that an interest in exploring the value of TXA in paediatric orthopaedic surgery is generated. Ideally this should be explored further in an adequately powered, randomised controlled trial where risk of bias is minimized.

## Figures and Tables

**Table 1:** The gross motor function classification system.

GMFCS	Descriptions
Level I	Children in this group can perform usual activities such as running and jumping almost as normal. There may be a decreased speed, balance, and coordination.

Level II	They have the ability to walk indoors and outdoors and climb stairs with a railing. They have difficulty with uneven surfaces, running, or jumping.

Level III	They need assistive mobility devices (such as canes, crutched, and walkers). They may be able to climb stairs using a railing.

Level IV	They use wheelchairs most of the time and may propel their own power wheelchair. They can participate in standing transfers.

Level V	There is severe limitation in all areas of motor function. They cannot sit or stand independently, even with adaptive equipment. They depend on others for mobility.

**Table 2:** Baseline characteristics of the study population.

Characteristics	No Tranexamic acid	Tranexamic acid	*P* value
Number	37	14	
Age (y)	10.2 (SD 3.3)	9.8 (SD 3.3)	0.74
Male (%)	21 (57%)	5 (36%)	0.22
Weight (kg)	30.2 (SD 11.8)	27.7 (SD 13.0)	0.54
GMFCS (low/high)	9/27	3/11	0.79
ASA (1/2/3/4/5)	3/18/8/0/0	1/6/5/0/0	0.32
Bilateral	10 (27%)	8 (57%)	0.06
Types of bony osteotomy (F, P, and B)	11/1/25	3/0/11	0.62
Prescribed NSAID (%)	19 (58%)	6 (50%)	0.74
Epilim (%)	5 (14%)	5 (36%)	0.12
Preoperative Hb (g/dL)	13.5 (SD 1.2)	13.3 (SD 1.4)	0.73
Preoperative Hct	0.397 (SD 0.032)	0.383 (SD 0.033)	0.19

GMFCS: gross motor function classification system, ASA: American Society of Anaesthetists, F: femoral osteotomy, P: pelvic osteotomy, B: both femoral and pelvic osteotomy, NSAID: nonsteroidal anti-inflammatory, Hb: hemoglobin, Hct: hematocrit, and SD: standard deviation.

**Table 3:** Primary and secondary outcomes.

	No tranexamic acid [1]	Tranexamic acid [2]	[2]−[1] (95% CI), *P*
Transfusion rate	13/36 (36.1%)	6/14 (42.9%)	6.7% (−21.3% to 36.1%), *P* = 0.75
Total blood loss, mL	971 (SD 766)	969 (SD 742)	−2 (−536 to 531), *P* = 0.99
Postop Hb, g/dL	9.4 (SD 1.76)	8.9 (SD 1.5)	−0.5 (−1.7 to 0.7), *P* = 0.40
Postop Hct	0.29 (SD 0.06)	0.27 (SD 0.04)	−0.02 (−0.06 to 0.02), *P* = 0.36
Hb drop, g/dL	4.1 (SD 1.9)	4.3 (SD 2.0)	0.2 (−1.1 to 1.6), *P* = 0.72
Length of stay, days	7.63 (SD 4.2)	8.17 (SD 3.9)	0.54 (−2.37 to 3.44), *P* = 0.71

Hb: hemoglobin, Hct: hematocrit, and SD: standard deviation.

**Table 4:** Adverse events.

Complications	No tranexamic acid	Tranexamic acid
Deep venous thrombosis	0	0
Pulmonary embolism	0	0
Deep infection	1	0
Paralytic ileus	0	1
Pressure sore	1	0
Fragility fracture	0	2
Total	2	3
